# Co-acting gene networks predict TRAIL responsiveness of tumour cells with high accuracy

**DOI:** 10.1186/1471-2164-15-1144

**Published:** 2014-12-19

**Authors:** Paul O’Reilly, Csaba Ortutay, Grainne Gernon, Enda O’Connell, Cathal Seoighe, Susan Boyce, Luis Serrano, Eva Szegezdi

**Affiliations:** Apoptosis Research Centre, National University of Ireland Galway, University Rd, Galway, Ireland; National Centre for Biomedical Engineering Sciences, National University of Ireland Galway, University Rd, Galway, Ireland; School of Mathematics, Statistics and Applied Mathematics, National University of Ireland Galway, University Rd, Galway, Ireland; HiDucator Ltd, Erämiehentie 2 E 22, 36200 Kangasala, Finland; School of Medicine, University College Dublin, Dublin 4, Ireland; EMBL/CRG Systems Biology Research Unit, Centre for Genomic Regulation (CRG), ICREA Professor, C/ Dr. Aiguader 88, 08003 Barcelona, Spain

**Keywords:** TRAIL, Biomarker, Gene expression, Random forest

## Abstract

**Background:**

Identification of differentially expressed genes from transcriptomic studies is one of the most common mechanisms to identify tumor biomarkers. This approach however is not well suited to identify interaction between genes whose protein products potentially influence each other, which limits its power to identify molecular wiring of tumour cells dictating response to a drug. Due to the fact that signal transduction pathways are not linear and highly interlinked, the biological response they drive may be better described by the relative amount of their components and their functional relationships than by their individual, absolute expression.

**Results:**

Gene expression microarray data for 109 tumor cell lines with known sensitivity to the death ligand cytokine tumor necrosis factor-related apoptosis-inducing ligand (TRAIL) was used to identify genes with potential functional relationships determining responsiveness to TRAIL-induced apoptosis. The machine learning technique Random Forest in the statistical environment “R” with backward elimination was used to identify the key predictors of TRAIL sensitivity and differentially expressed genes were identified using the software GeneSpring. Gene co-regulation and statistical interaction was assessed with q-order partial correlation analysis and non-rejection rate. Biological (functional) interactions amongst the co-acting genes were studied with Ingenuity network analysis. Prediction accuracy was assessed by calculating the area under the receiver operator curve using an independent dataset. We show that the gene panel identified could predict TRAIL-sensitivity with a very high degree of sensitivity and specificity (AUC = 0 · 84). The genes in the panel are co-regulated and at least 40% of them functionally interact in signal transduction pathways that regulate cell death and cell survival, cellular differentiation and morphogenesis. Importantly, only 12% of the TRAIL-predictor genes were differentially expressed highlighting the importance of functional interactions in predicting the biological response.

**Conclusions:**

The advantage of co-acting gene clusters is that this analysis does not depend on differential expression and is able to incorporate direct- and indirect gene interactions as well as tissue- and cell-specific characteristics. This approach (1) identified a descriptor of TRAIL sensitivity which performs significantly better as a predictor of TRAIL sensitivity than any previously reported gene signatures, (2) identified potential novel regulators of TRAIL-responsiveness and (3) provided a systematic view highlighting fundamental differences between the molecular wiring of sensitive and resistant cell types.

**Electronic supplementary material:**

The online version of this article (doi:10.1186/1471-2164-15-1144) contains supplementary material, which is available to authorized users.

## Background

Tumor necrosis factor-related apoptosis-inducing ligand (TRAIL, TNFSF10), a member of the TNF cytokine family, is an emerging therapeutic option for various cancers. Due to the tumor-specific cytotoxicity of TRAIL, its recombinant version and agonistic antibodies against the death-inducing TRAIL receptors (TNFRSF10A/DR4, TNFRSF10B/DR5) are currently being tested in Phase I/II clinical trials. The basis for the tumor-specific action of TRAIL is that during malignant transformation cells become sensitive to TRAIL [1]. During later progression however, tumors can re-acquire resistance to evade immune-mediated killing and thus, prediction of tumor TRAIL-responsiveness is critical [2]. While TRAIL can be a very potent tumoricidal agent due to its ability to target both the tumor cells and the tumor vasculature [3], administration of TRAIL to resistant tumors may trigger invasiveness and promote metastasis [4–6] further highlighting the need for robust biomarkers predicting TRAIL-responsiveness. While the TRAIL-induced apoptotic machinery is well studied and a number of regulatory mechanisms have been identified, none of them have proven to be useful as a predictive marker.

Owing to its high sensitivity and full coverage of the human genome, transcriptomics is one of the most widely applied tools for biomarker research by selecting genes that are differentially expressed in the majority of the samples, or in a specific subgroup. This gene-by-gene approach however cannot address the question of how genes relate to each other in determining the biological outcome. Often the change in the expression of key regulatory genes is minor, or it is only significant in a small subset within a diverse sample cohort, or its effect on the phenotype is conditional on the expression level of other genes. Thus the relative expression of genes acting/participating in the same biological process and their combinatorial analysis may better describe the behavior of a cell and predict the response to a stimulus.

Here we show that it is possible to predict TRAIL sensitivity with high accuracy by using hierarchical decision tree analysis of transcriptomic data. We found that the identified predictor genes are not differentially expressed, but instead they have linked biological functions (including inhibition, activation, induction of expression etc.). We report here that these “co-acting” gene clusters can be identified from transcriptomic data and these co-acting genes could predict TRAIL-responsiveness with a much higher degree of sensitivity and specificity (AUC = 0.84) than the currently available best-performing gene signature predicting TRAIL-responsiveness (AUC of 0.72) [7].

## Results

The focus of the study was to identify genes that predict TRAIL-responsiveness and analyse their correlation and functional interactions, such as regulation by direct interaction, post-translational modification, induction or repression of expression, induction of degradation etc. (analysis workflow is shown in Additional file 1: Figure S1).

**Figure 1:**
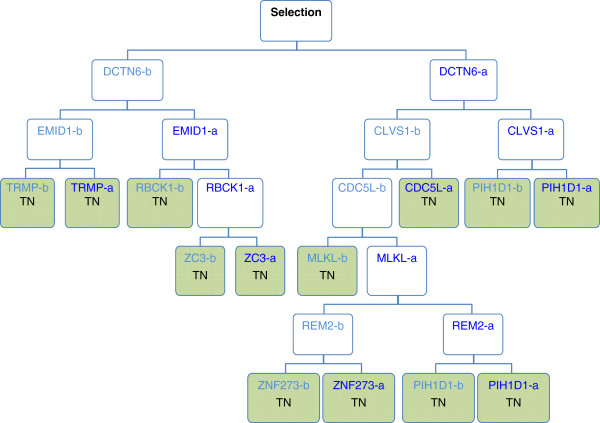
**Depiction of a classification tree.** For a tree within the random forest a bootstrap or subset of cell lines (samples) and genes (variables) is randomly selected. The gene from this random subset whose expression best divides the cell lines as cleanly as possible into sensitive and resistant is then selected. In the example above this gene was DCTN6. The two groupings or daughter nodes are not pure, they have some resistant and some sensitive cell lines, so a new subset of genes is selected and the gene which best divides the cell lines is selected; in the example above these genes were EMID1 and CLVS1. This recursive partitioning continues until terminal nodes (TN; green colored nodes) are reached, these being groupings in which only sensitive or resistant cell lines are found. In this manner classification trees consider the expression of one gene in relation to another (“co-acting genes”) in determining the biological response (i.e. TRAIL-sensitivity).

First, each gene was mapped to a singular “best” probeset using the JetSet algorithm as described in the Materials and methods [8]. Expression values of the selected 19,190 probesets were used to identify genes that predict TRAIL-sensitivity by growing decision tree ensembles in the Random Jungle implementation of the Random Forest (RF) algorithm [9, 10]. The algorithm generates a large number of independent decision trees each containing a random subset of the samples and a random subset of the predictors (genes). Each decision tree branches downward by repeated selection of genes that best separate the samples, until the sample subset is fully divided into homogeneous groups (sensitive or resistant) as shown in Figure 1. Random forests are not limited by the need of a gene to be differential expressed but can include gene interactions, as the gene used to partition the data at a node can be influenced by the genes at higher levels of the tree. We refer to the group of such genes as co-acting.

**Figure 2:**
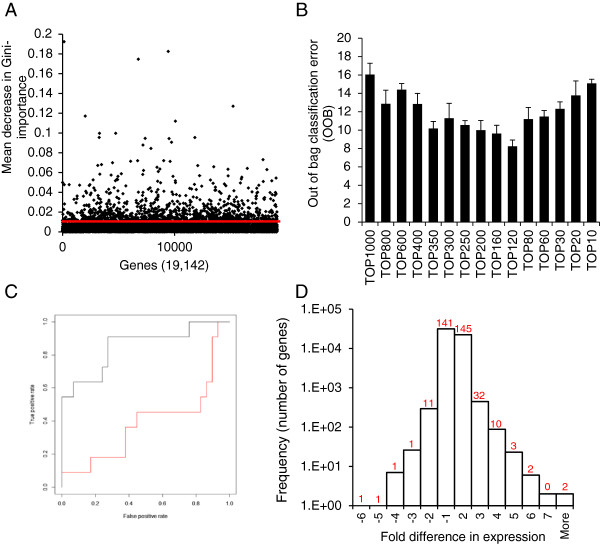
**Identification of the core co-acting gene set. (A)** Gene ranking by Gini-importance. A singular “best” probeset for each gene was used to grow 10,000 classification trees. The importance of each gene in classifying cell lines as sensitive or resistant to TRAIL was measured by mean decrease in Gini-importance in the training dataset. The probesets above the red line represent the top 5^th^ percentile retained for further analysis. Only genes with Gini-importance value higher than zero were plotted. **(B)** The top 350 genes predict TRAIL-responsiveness with high accuracy. From the top-ranked 1000 genes, the lowest ranked genes were stepwise removed (by units of 100 and then 10) and the performance of the remaining gene-set was determined by calculating the out of bag classification error (OOB) (stepwise 10-gene unit removal between top 300-top 200 genes had no effect and thus it is not shown on the graph). **(C)** Validation of the prediction accuracy of the 350 co-acting genes. The area under the receiver operator curve (AUC) was calculated as a measure of the models specificity and sensitivity in the independent test dataset on the dataset (black line, AUC = 0 · 85) as well as after swapping the sensitivity values of a randomly-selected 50% of the cells lines (red line, AUC = 0 · 48). The graph shows the AUC. This is a representative graph from 100 repeats of random permutations. **(D)** The 350 co-acting genes are not identified by differential expression analysis. A histogram displaying the gene distribution based on fold difference in expression between TRAIL sensitive and resistant cell lines. The number of genes from the panel of 350 co-acting genes falling in the individual fold difference ranges on the histogram is indicated by the numbers above each column.

The importance of the 19,190 genes in predicting TRAIL-responsiveness was determined by calculating the mean decrease in Gini-importance, which is based on calculating the reduction in prediction accuracy after permuting the expression value of the gene in question (referred to as Gini-importance). From the Gini-importance list the top fifth percentile (the highest ranking 1000 genes) was retained for further analysis (Figure 2A). These genes could predict TRAIL-responsiveness with an out of bag (OOB) error of 16%. To improve the performance of the model, the bottom-ranking genes of the Gini-importance list were stepwise removed (backward elimination), the RF model rerun and the performance assessed by calculating the OOB error (Figure 2B). This analysis identified that the top 350 as well as the smaller subset of the top-ranking 120 genes, performed best with OOB error rates of 10 · 1% and 8 · 3%, respectively. Since the contribution and importance of individual genes is likely to be different in different sample types, the larger, 350 gene subset was chosen as the TRAIL-response predictor co-acting gene panel (listed in Additional file 2: Table S1).An independent dataset (NIH CellMiner) was used to determine the prediction accuracy of the 350 gene-panel and it confirmed that these genes predicted TRAIL-responsiveness with high accuracy of 0.84, measured as the area under the receiver operator characteristic curve (AUC of ROC curve, Figure 2C). To test the relevance of these genes as predictors of TRAIL-responsiveness, the sensitivity value (sensitive or resistant) of the cell lines was changed to the incorrect alternative in a randomly selected 50% of the samples and the accuracy of the model determined. The AUC reduced to 0.48 (p < 0 · 05) confirming that the prediction accuracy achieved was unlikely to have occurred by chance (Figure 2C).The genes differentially expressed between sensitive and resistant cell lines were then identified and compared to the co-acting gene panel identified with RF. There were 254 genes that showed a minimum of 2-fold difference in expression and were considered statistically significant. Interestingly, the majority (82%) of the co-acting genes were not differentially expressed (Figure 2D).

**Figure 3:**
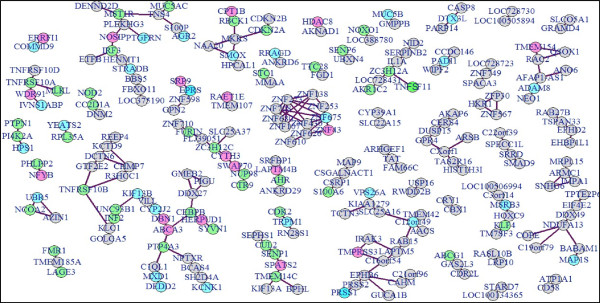
**Co-regulated gene clusters.** Relationship between gene pairs was assessed using q-order partial correlation and gene clusters passing non-rejection rate threshold of 0.075 at q = 35 were graphed using qpgraph package in R. Green coloured gene nodes represent identified components of cell death and cell survival signal transduction pathways depicted in Figure 5, blue nodes of components of the cellular- and embryonic development network shown in Additional file 2: Figure S2 and pink nodes showing genes within the cancer-related and reproductive system network depicted in Additional file 3 Figure S3.

In order to assess whether the genes selected with RF are co-regulated q-order partial correlation with non-rejection rate (NRR) thresholding was carried out. The analysis showed strong correlation amongst the genes; even at a stringent non-rejection rate threshold of 0 · 15 (at q = 35) over 90% of the genes had at least one interacting gene partner (Table 1). The most significant co-regulated gene clusters were identified by graphing the co-regulated gene network from these associations at decreasing NRR threshold until individual gene clusters separated out (q = 35, NRR = 0 · 075, Figure 3).

**Table 1:** **The percentage of genes which had at least one co-regulated gene partner**

	Q value		
NRR	1	35	70
**1**	100	100	100
**0.8**	100	100	100
**0.6**	100	100	99
**0.4**	100	100	57
**0.2**	100	97	26
**0.15**	100	93	19
**0.1**	100	81	13
**0.05**	100	59	9
**0.01**	100	33	5
**0.005**	100	28	5

The percentage of genes which had at least one co-regulated gene partner within the 350 co-acting gene panel determined by q-order partial correlation at different non-rejection rate thresholds.

**Figure 4:**
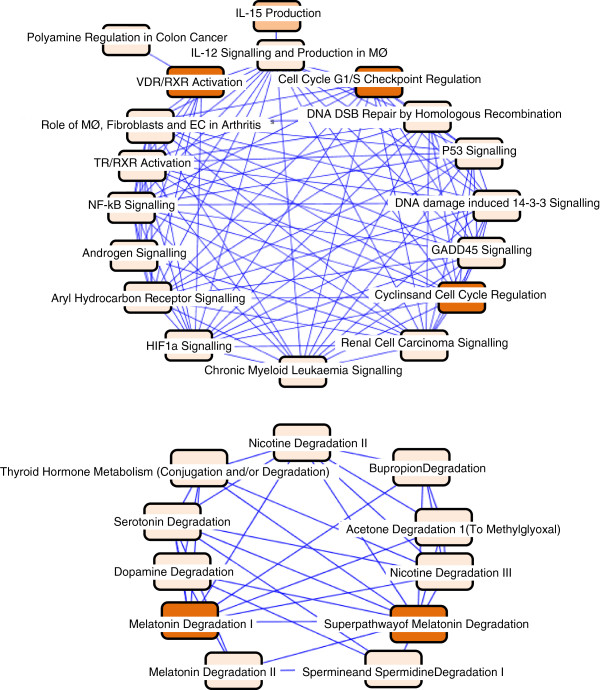
**The Canonical pathways the 350 co-acting genes are involved in.** The canonical signal transduction pathways and their interactions that the 350 co-acting genes function within. The intensity of the box colour is indicative of statistical significance as determined by Fishers exact test. DSB: double strand break, EC: endothelial cell, GADD45: growth arrest and DNA damage-inducible 45, IL: interleukin, MØ: macrophage, NF-kB: nuclear factor kappa B, RXR: retinoid X receptor, TR: thyroid receptor, VDR: vitamin D receptor.

Finally, biological functional network analysis was carried out to determine whether the protein products of the co-regulated genes interact *in vivo*, within the cell. Multiple databases of experimentally proven biological interactions (such as induction, binding, activation, inhibition as well acting within the same canonical signaling pathway) were searched using the Ingenuity Pathway Analysis Platform (Ingenuity, IPA, Qiagen). The analysis showed that the 350 genes are enriched in several highly interconnected canonical signal transduction pathways, which were focused around 5 major themes: 1. Cell cycle and DNA damage, 2. Nuclear receptor signaling, 3. Neurotransmitter metabolism/signaling 4. Inflammatory/immune reaction and 5. Cancer tissue-specific pathway transformation (Figure 4).

**Figure 5:**
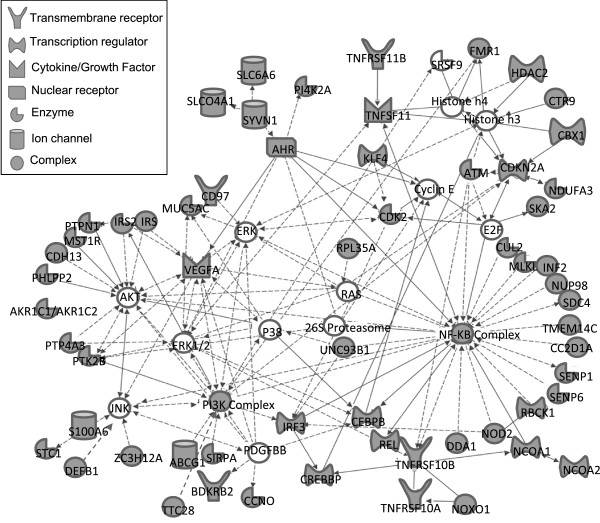
**The genes determining TRAIL-response-tend to be upstream regulators of components of cell death and cell survival signal transduction pathways.** The figure shows direct (solid lines) and indirect interactions (dashed lines) amongst components of the cell death and survival signal transduction pathways. Genes from the 350 gene panel are coloured grey. Lines with arrowheads indicate functional interaction, such as regulation of expression or activity, while lines without arrowheads indicate protein-protein interactions.

Analysis of biological interactions showed that over 40% of the genes (141 in total) were proven or predicted components of three interconnected signaling networks controlling (1) cell death and pro-survival signal transduction (65 genes, Figure 5), (2) cellular differentiation and morphogenesis (45 genes, Additional file 3: Figure S2), and (3) cancer related signaling pathways (31 genes, Additional file 4: Figure S3). The remaining genes could not be grouped into signaling networks based on the current literature about them in the searched databases. These networks confirm that interactions between the 350 genes exist at the biological level. Notably, in the network of *cell death and pro-survival signaling pathways*, the majority of the 350 genes are upstream regulators of protein kinases and transcription factors, such as NF-κB (nuclear factor-kappa B), PI3K (phosphatidylinositol 3-kinase), JNK (c-Jun N-terminal kinase), and ERK (extracellular signal-regulated kinase) (Figure 5).

**Figure 6:**
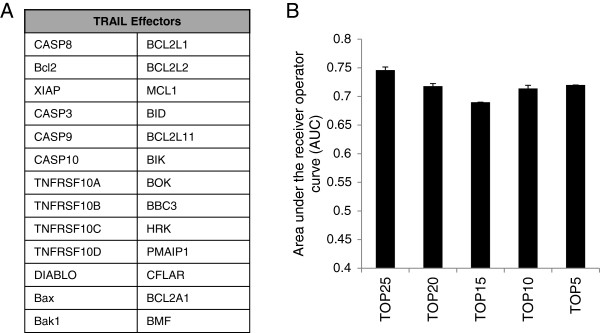
**The known core components and regulators of the TRAIL apoptotic machinery do not predict TRAIL sensitivity with a high degree of sensitivity and specificity. (A)** The selected 26 core component genes and regulators of the TRAIL apoptotic. **(B)** Performance of the 26 “TRAIL effectors” as a classifier assessed using the same approach as for the whole transcriptome (workflow shown in Additional file 1: Figure S1). The 26 core effectors predicted TRAIL sensitivity poorly (AUC = 0.46). Removal of any of the core effectors caused a decrease in the model’s ability to predict sensitivity to TRAIL.

By contrast, in the *cancer-related* and the *cell differentiation and morphogenesis* networks the TRAIL-predictor genes are mostly target genes, rather than upstream regulators, under the control of proteins typically functioning in chromatin remodeling and transcriptional regulation. The main examples include nuclear protein 1 (NUPR1, a binding partner for p53 and the estrogen receptor with a multifaceted role in tumorigenesis), p53, the DNA helicase SMARCA4 (alters chromatin structure for transcription activation), lysine-specific demethylase 5B (KDM5B), estrogen receptor, heat shock factor-1 (HSF-1, transcription factor for stress-mediated heat shock protein induction), and bromodomain-containing protein 4 (BRD4, chromatin reader protein) (Additional file 3: Figure S2 and Additional file 4: Figure S3).The components and regulators of TRAIL signal transduction are considered to be well studied and understood. We identified the 26 core effectors of TRAIL-mediated apoptosis signaling from the literature (Figure 6A) and determined whether the inter-relationships between these genes using the RF model would predict TRAIL-sensitivity. We found that the prediction accuracy of the 26 core effectors was inferior compared to the 350 co-acting gene set (AUC = 0 · 74) and backward elimination by mean decrease in Gini-importance only worsened the prediction suggesting that genes not in this core set are likely to be important for predicting TRAIL-responsiveness (Figure 6B).

## Discussion

Biomarkers are pillars of diagnostic biology both for detection and prognosis. In the last number of years there has been a paradigm shift from the identification of prognostic and diagnostic biomarkers to biomarkers that can predict treatment efficacy. This refocusing has been facilitated by the advent of high-throughput technologies such as genome-wide association analysis, transcriptomics or metabolomics and already resulted theranostic diagnostics, such as the Oncotype-DX biomarker panel for treatment-identification for breast cancer patients [11]. Differential gene expression patterns from transcriptome studies can be also used to identify drugs that have the potential to reverse an unfavorable phenotype, such as drug-resistance with the help of the computational algorithm Connectivity mapping (Broad Institute of Massachusetts Institute of Technology, Harvard University) [12].

Biomarkers to predict the functionality of the TRAIL apoptotic pathway in cancer cells are becoming increasingly important with emerging promising phase I trials and new pre-clinical studies showing the potency of TRAIL on the tumor vasculature and synergistic DR5-activation by the combination of TRAIL and agonistic DR5 antibody (AMG655, Amgen) in ovarian cancer [3, 13]. Some of the pathway regulators have been indicated as potential biomarkers, including GalNT14, c-FLIP, DcR1 or DcR2 as individual markers [14–19] or groups of differentially expressed genes [7]. Currently the best classifier of TRAIL sensitivity is a 71-gene signature of genes differentially expressed between TRAIL-resistant and sensitive tumor cell lines with a prediction accuracy of AUC = 0 · 72 [7].

Here we show that by using machine-learning methods we could predict the TRAIL-responsiveness of cancer cell types with an accuracy superior to any of the current markers. Importantly, most of the identified genes were not differentially expressed; instead 40% of them were linked based on their biological function.

The advantage of the co-acting gene clusters in prediction can be due to their ability to capture the non-linear nature of signal transduction pathways, which one-dimensional analyses, such as differential expression poorly reflect. It is also well established that not all cells utilize the same mechanism to block apoptosis [1, 19]. Ensembles of decision tree models can follow and identify branching gene interactions and are able to simultaneously test a number of potential routes of co-acting gene linkages that describe the cell’s phenotype even in highly diverse sample sets. Finally, unlike differential expression analyses, co-acting gene clusters do not exclude genes which are differentially expressed only in a small subset of a diverse sample population.

In comparison, the well-documented components of the TRAIL pathway were poor predictors (AUC = 0.74). Complementing this, out of the 350 co-acting genes only six, namely DR4, DR5, DcR2, osteoprotegerin, Caspase-8, and heme-oxidized IRP2 ubiquitin ligase 1 (HOIL1) are well characterized components of TRAIL signaling (Additional file 2: Table S2). Another 14 genes linked to TRAIL-sensitivity by at least one study are present in the panel, including mixed-lineage kinase domain-like (MLKL), DNA-binding death effector domain-containing protein 2 (DEDD2), NADPH oxidase organizer 1, the serine/threonine kinase Ataxia telangiectasia mutated (ATM), polypeptide N-acetylgalactosaminyltransferase-14 and cyclin-dependent kinase 2 (please refer to Additional file 2: Table S2 for full list). The remaining 336 genes have not been associated with TRAIL. These genes may be co-regulated with other genes that regulate TRAIL sensitivity, but have no effect on the pathway (bystanders), although it is more likely that many of them directly or indirectly regulate TRAIL-sensitivity.

IPA functional pathway analysis revealed that 40% of the identified genes have already been reported to interact either directly or indirectly. Many co-acting genes are upstream regulators of well-documented regulators of the TRAIL signaling machinery, such as NF-κB, p53, or AKT. In addition to these known TRAIL-signaling regulators, other nodal points in the signaling networks were proteins that have established roles in cancer progression and/or resistance to cytotoxic drugs but have not been implicated in TRAIL-induced apoptosis previously. For example KDM5B (Histone demethylase JARID1B) is overexpressed in many cancers including breast, prostate, and lung cancer as well as melanoma where it confers resistance to apoptotic stimuli such as cisplatin and vemurafenib [20, 21]. Recent reports suggest that KDM5B functions through E2F1/2, which is known to be able to modulate sensitivity to TRAIL [22, 23]. Another example is NUPR1 (Nuclear protein 1), known to be highly expressed in a wide range of cancerous malignancies and its expression has been inversely correlated with the induction of apoptosis by various compounds such as doxorubicin [24, 25].

## Conclusions

We show that a random forest classifier based on gene expression performs significantly better than previously reported biomarkers in predicting sensitivity to TRAIL in tumor cell lines perhaps because it allows a more flexible description of the molecular networks present in individual cells or cell types. These findings shed light on why previous studies failed to find a reliable marker of TRAIL sensitivity and also pinpoint potential novel regulators of the pathway.

## Materials and methods

### Analysis of microarray data

Raw transcriptome microarray data for 109 cell lines from Gene Expression Omnibus (GEO) accession number GSE8332 (training dataset) [26] and for an additional 40 tumor cell lines (NIH CellMiner, test dataset) was acquired in CEL file format. Background correction and normalization of the datasets has been performed using the RMA algorithm in the Affy package of Bioconductor [27] in the statistical environment, R (version 2.10.1). Noise in the analysis caused by multiple probesets per gene was reduced by identifying the probeset best representing each gene based on scoring and ranking the probesets for specificity, splice isoform coverage and robustness against degeneration using the JetSet algorithm [8].

Differentially expressed genes were identified using Genespring GX v11. After importing the CEL files, gene expression values were transformed (Log_2_) and normalized to the 75^th^ percentile using the RMA algorithm. For each cell line the genes whose expression was above the expression value of the 20^th^ percentile across all cell lines were selected. These genes were filtered by retaining only those which were present in at least 75% of the cell lines *and/or* exhibited a greater than 2 fold change in expression between TRAIL sensitive and resistant cell lines. One-way ANOVA unequal variance (Welch) was used to test for significance using a cut-off value of p ≤ 0.05. Multiple testing corrections were done by Benjamini-Hochberg.

### Determining TRAIL sensitivity of cancer cell lines

TRAIL sensitivity of the cell lines was assessed and described by Wagner and colleagues using MTT assay [26]. A cell line was defined as being sensitive if 1 ug/ml recombinant human TRAIL (rhTRAIL) reduced viability to 50% within 72 h or less and resistant if the reduction in viability was less than 50%.

### Classification of tumor cell lines

Jetset-identified mRNA probesets for each gene were extracted from the training microarray data and were used for classifying the cell line samples into TRAIL-sensitive and resistant groups using random jungle, a parallel implementation of random forest (RF) modeling reducing computational time for high dimensional datasets (number of classification trees = 10,000, mtry = default). In order to avoid imbalance between the number of sensitive and resistant cell lines during training, the sample number to grow the trees was set to 30 sensitive and 30 resistant cell lines and the remaining samples were used to determine the OOB error rate (sampsize). The genes were ranked by mean decrease in Gini-importance. In order to determine the core co-acting gene-set, the lowest ranking genes were iteratively removed and the model’s performance tested by determining out of bag (OOB) error rate. Performance of the final model was measured by predicting TRAIL-responsiveness of the test dataset and calculating the area under the receiver operator curve (AUC) from 10 repeats. All statistical analyses were carried out using Random Jungle [9] and RandomForest [10] packages in the R (version 3 · 0 · 3) environment (http://www.r-project.org/).

### Gene relationship analysis

The strength of the statistical interactions between genes was measured with q-order partial correlation using the qpgraph package in the Bioconductor project in R (version 3 · 0 · 3) [28]. This analysis determines interaction between two genes while filtering out potential effects of other genes (number of genes for filtering is the q value). Non-rejection rates were calculated from the normalized gene expression data using q values ranging between 1–105 q-order partial correlations. Gene regulatory networks where nodes represent genes and edges represent the partial correlation between the genes were graphed using the igraph package in R (version 3 · 0 · 3).

### Ingenuity pathway analysis (IPA)

Biological interactions and inter-relationship amongst the co-acting genes were analyzed using the Ingenuity IPA network analysis tool (Qiagen) by performing core analysis with the following settings: reference set: human genome U133 plus 2 · 0 Array, included both direct and indirect relationships experimentally observed limited to human with the maximum number of network components set to 140 genes. Statistical significance for canonical pathway and network analysis was determined by Fisher’s exact test.

## Electronic supplementary material

Additional file 1: Figure S1: Workflow of gene expression analysis. (JPEG 551 KB)

Additional file 2: Table S1: Genes of the 350 co-acting gene classifier panel. Bold highlighted genes are known components or regulators of TRAIL-sensitivity. **Table S2.** Genes of the 350 co-acting gene panel with known association with TRAIL sensitivity. (DOCX 58 KB)

Additional file 3: Figure S2: TRAIL-predictor genes interact in cancer-related signal transduction networks. (JPEG 523 KB)

Additional file 4: Figure S3: TRAIL-predictor gene product are interacting proteins of cellular differentiation and morphogenesis regulatory pathways. (JPEG 547 KB)
