# Supplementary figures and images for: Co-acting gene networks predict TRAIL responsiveness of tumour cells with high accuracy
Source: BMC Genomics. 2014 Dec 19;15(1):1144. doi: 10.1186/1471-2164-15-1144 (PMC4378270; doi:10.1186/1471-2164-15-1144)

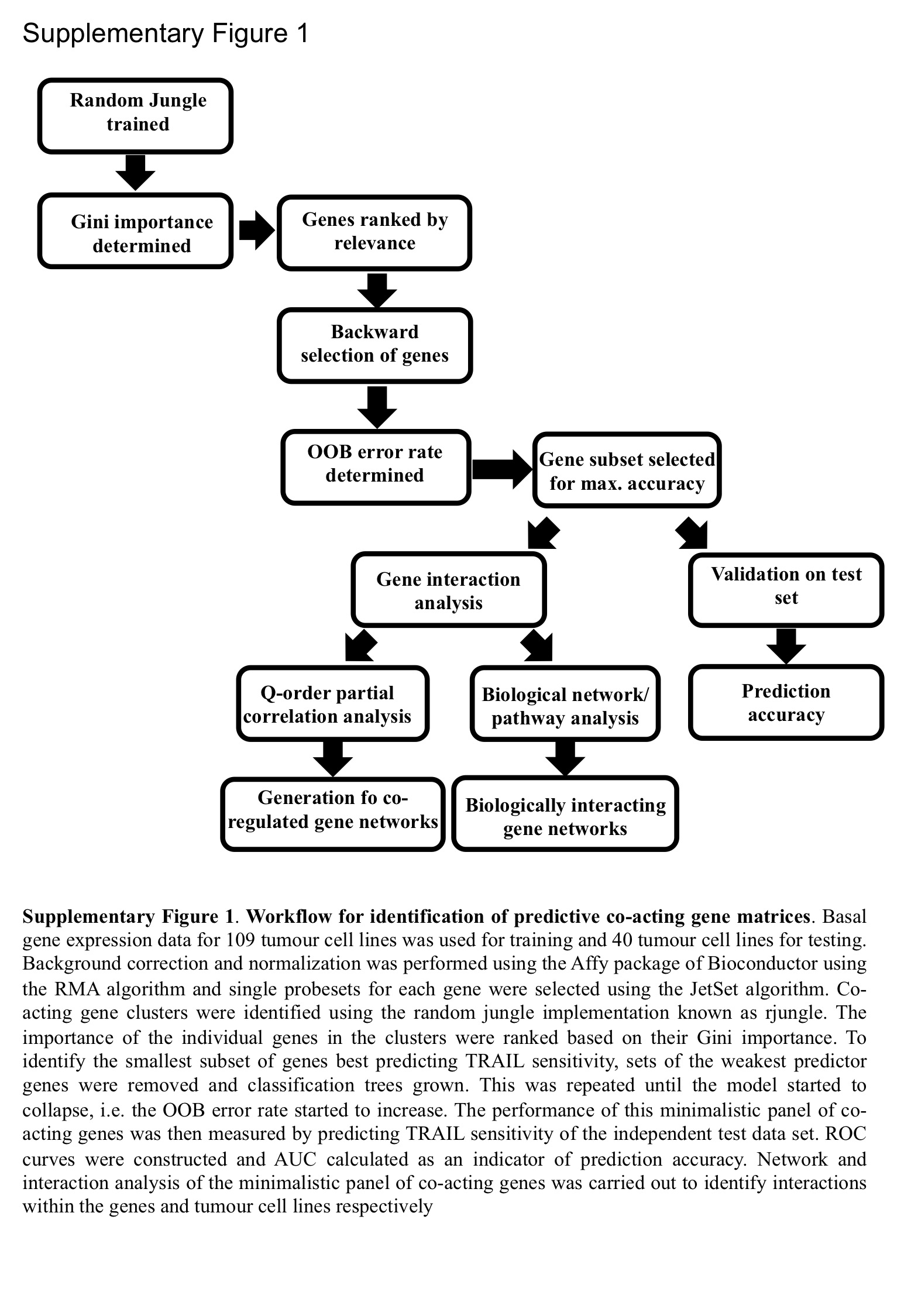

Supplement: Supplementary file 1 — Additional file 1: Figure S1: Workflow of gene expression analysis. (JPEG 551 KB) [file 12864_2014_6883_MOESM1_ESM.jpeg]

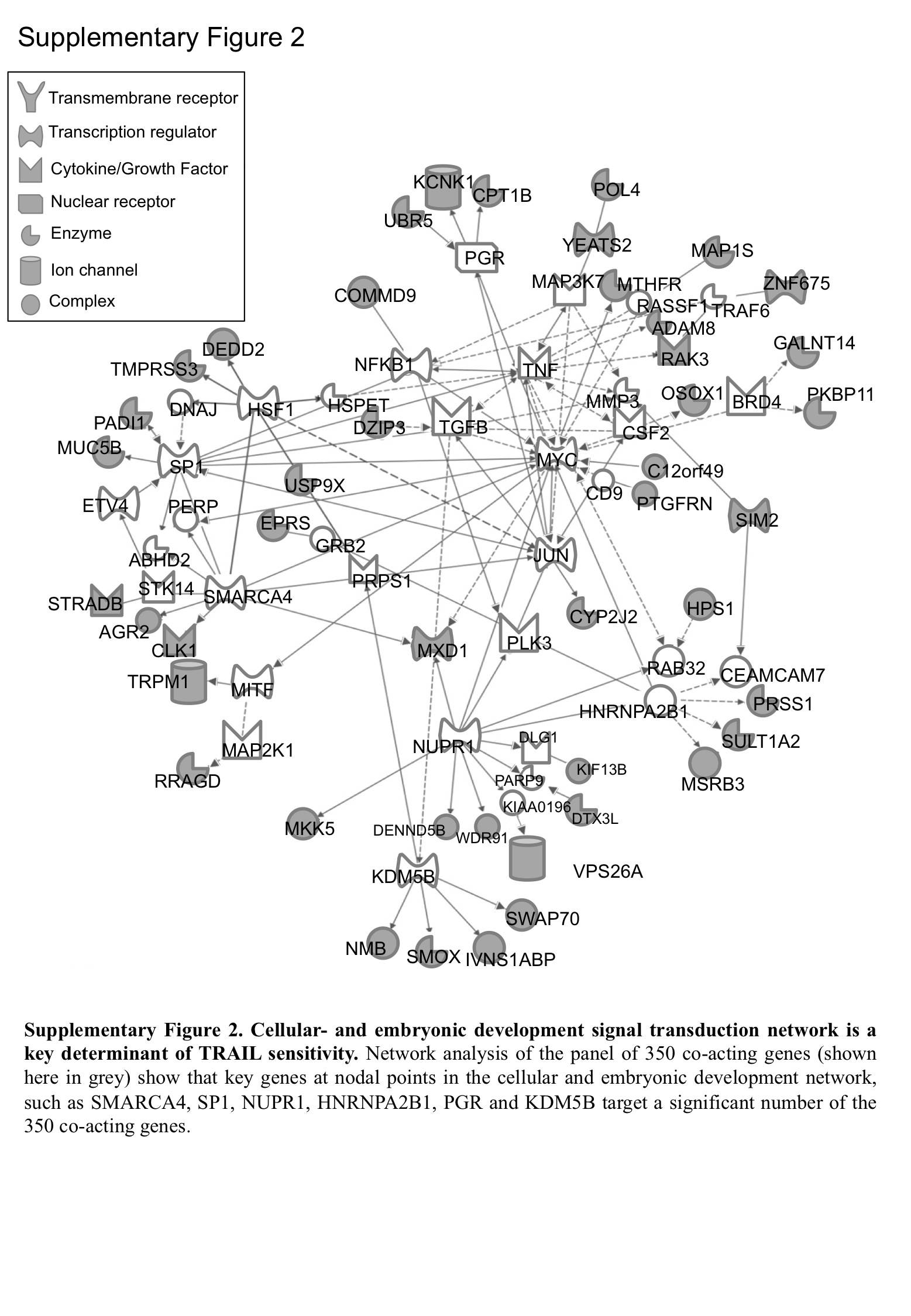

Supplement: Supplementary file 3 — Additional file 3: Figure S2: TRAIL-predictor genes interact in cancer-related signal transduction networks. (JPEG 523 KB) [file 12864_2014_6883_MOESM3_ESM.jpeg]

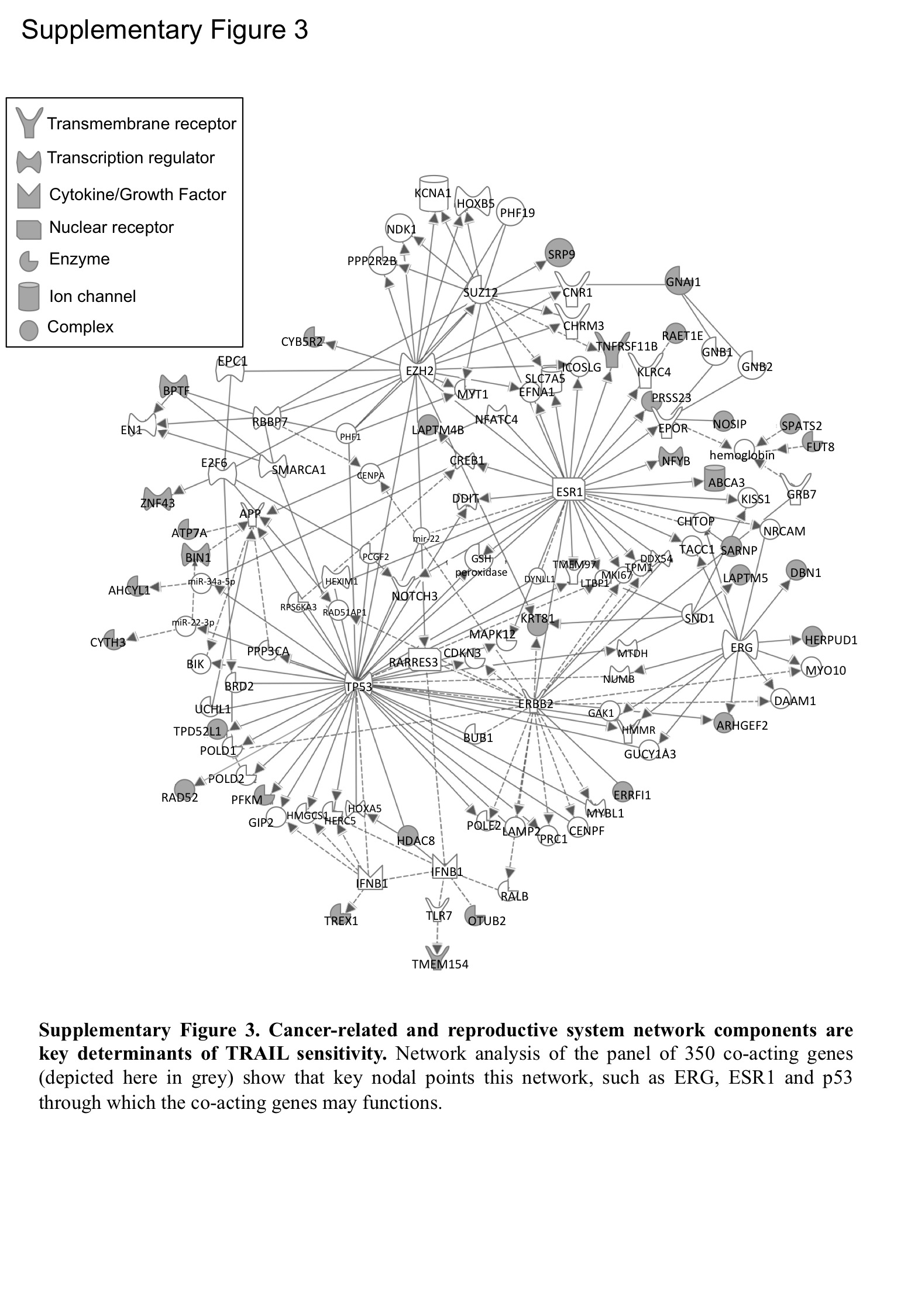

Supplement: Supplementary file 4 — Additional file 4: Figure S3: TRAIL-predictor gene product are interacting proteins of cellular differentiation and morphogenesis regulatory pathways. (JPEG 547 KB) [file 12864_2014_6883_MOESM4_ESM.jpeg]
