# Supplementary material for: Co-acting gene networks predict TRAIL responsiveness of tumour cells with high accuracy
Source: BMC Genomics. 2014 Dec 19;15(1):1144. doi: 10.1186/1471-2164-15-1144 (PMC4378270; doi:10.1186/1471-2164-15-1144)
Supplement: Supplementary file 2 — Additional file 2: Table S1: Genes of the 350 co-acting gene classifier panel. Bold highlighted genes are known components or regulators of TRAIL-sensitivity. Table S2. Genes of the 350 co-acting gene panel with known association with TRAIL sensitivity. (DOCX 58 KB) [file 12864_2014_6883_MOESM2_ESM.docx]

| Additional file 2: Table S1. Genes of the 350 co-acting gene classifier panel. Bold highlighted genes are known components or regulators of TRAIL-sensitivity. | | | | | | | | | |
| --- | --- | --- | --- | --- | --- | --- | --- | --- | --- |
| TNFRSF10A | MLKL | BIN1 | FURIN | ARSB | SYNC | CASP9 | PCNXL2 | SLCO5A1 | NDUFAF1 |
| PIH1D1 | ZNF273 | KRT81 | GPR108 | DUSP19 | UBR4 | RWDD2B | MAP1S | ZNF675 | D4S234E |
| TNFRSF10B | AGR2 | GPR4 | AKAP6 | ZNF491 | S100P | CASP8 | CERS4 | LOC100505894 | S100A6 |
| DENND5B | RBCK1 | IMPA1 | GPN2 | CDKN2B | KLC1 | CASP4 | MMAA | VEGFA | LAGE3 |
| CSRP1 | INF2 | CPT1B | SLC25A37 | PSMA2 | PION | ZNF610 | QSOX1 | ECH1 | ARSD |
| ZC3H12A | TNFRSF11B | EPHB6 | RAC2 | TNFRSF10D | HENMT1 | IL1A | TTC30A | CCDC146 | TAS2R16 |
| ETFB | DCTN6 | NAA20 | TMEM154 | ZNF253 | STK11IP | R3HCC1 | ZNF138 | NFYB | ZC3H12C |
| PRSS2 | GTF2E2 | SMAD9 | RN28S1 | C21orf56 | DNM2 | KIAA0889 | CCNO | FAM66C | CERS1 |
| CD97 | ARMC1 | FAM13B | KCTD9 | TSPAN33 | RAB27B | SERPINB2 | CYB5R2 | CEP78 | FHDC1 |
| KLF4 | SNHG6 | NCOA2 | MUC5AC | CD58 | ZNF107 | GAS2L3 | PTPN1 | HDAC8 | MSRB3 |
| TNFSF11 | KCTD7 | TNS4 | BPTF | CHMP7 | HPCAL1 | AHCYL1 | OTUB2 | KIAA1279 | PARP14 |
| EFHD2 | SNX12 | ZNF710 | DEDD2 | LDLR | ZNF749 | PWP1 | PACRGL | NOSIP | SIRPA |
| SPATS2 | SLC25A16 | TSPAN3 | STARD9 | HOXC9 | ACADVL | SENP1 | C16orf54 | DNM3 | LOC100506994 |
| HPS1 | CYP4X1 | C1QL1 | EHBP1L1 | ZNF626 | TMEM42 | GALC | FLJ39051 | CYP39A1 | SLCO4A1 |
| SPECC1L | ZNF708 | CYP27B1 | LRP10 | ATP1A1 | KRCC1 | TRPM1 | FAM53A | TMCO4 | CAHM |
| RPL35A | TMEM107 | TM7SF3 | CYTH3 | DENND2D | KIF13B | LNX1 | FAM132B | RIMS2 | DZIP3 |
| RRAGD | BDKRB2 | ANO6 | AACS | NUP98 | COMMD9 | MRPL15 | TREX1 | PADI1 | DEFB1 |
| USP35 | SPTLC3 | VILL | CSGALNACT1 | TTC28 | AZIN1 | CDKN2A | WDR91 | SARNP | LOC375190 |
| TMEM185A | GMPPB | STARD7 | ZNF43 | LAPTM4B | AKR1C2 | KIF13A | BABAM1 | MTHFR | CC2D1A |
| GUCA1B | NPTXR | CDK2 | MXD1 | MST1R | NFXL1 | IVNS1ABP | UBXN4 | ARHGEF1 | PIGU |
| GALNT14 | REL | USP9X | SENP6 | ABCA3 | FGD1 | NDUFA13 | IRS2 | NEO1 | FBXO11 |
| C21orf96 | SORBS2 | SYVN1 | FMR1 | C12orf49 | LOC728431 | CEBPB | C19orf79 | GRAMD4 | TBC1D8 |
| PHLPP2 | NUDT9P1 | SKA2 | SULT1A2 | PTK2B | C3orf14 | ANKRD6 | USP16 | DDX27 | PI4K2A |
| ABCG1 | CLK1 | KLHL13 | HIST1H3I | SH2D4A | LOC728723 | ZNF567 | SMOX | BPHL | RAB15 |
| ILVBL | PTGFRN | NOD2 | TAT | TPD52L1 | DDA1 | VPS26A | MAP9 | SRRD | AHR |
| HERPUD1 | CBX1 | WIPF2 | HDAC2 | MKKS | STRADB | XDH | CDH13 | TCTN3 | LAPTM5 |
| POLE4 | REEP4 | LOC388780 | CATSPERB | ARMC4 | LOC728730 | GOLGA5 | C1orf115 | C9orf125 | PAQR5 |
| SERPIND1 | SRFBP1 | SDC4 | FKBP11 | SEPHS1 | BBS5 | CEPT1 | NMB | COPE | ATM |
| ZNF430 | CUL2 | EML2 | CRY1 | PRSS1 | PLEKHG3 | RAET1E | SRP9 | NPAS2 | KCNK1 |
| GMEB2 | C14orf37 | UNC93B1 | GALK1 | AKNAD1 | GNAI1 | FUT8 | ATP7A | NCOA1 | AFAP1-AS1 |
| MUC5B | LOC100134365 | UBR5 | ZFP30 | IRF3 | TMPRSS3 | PRSS23 | LRRFIP1 | CYP2J2 | PTP4A3 |
| DBN1 | KIAA1467 | SPACA3 | C22orf39 | CXorf1 | DHRS3 | C11orf95 | ADAM8 | IRAK3 | DTX3L |
| ANKRD29 | SLC6A6 | HKR1 | BCAS4 | TPTE2P6 | NID2 | ERRFI1 | SLC22A15 | SIM2 | CDR2L |
| LOC100287765 | TMEM14C | DUSP15 | ZNF680 | NOXO1 | CREBBP | STC1 | EPRS | VPS41 | CTR9 |
| RASL10B | DDX49 | EIF4E2 | PFKM | YEATS2 | LY6G5C | SWAP70 | ZNF598 | CECR6 | RAD52 |

| **Additional file 2: Table S2. Genes of the 350 co-acting gene panel with known association with TRAIL sensitivity** | |
| --- | --- |
| **Co-acting gene** | **Reference** |
| Mixed lineage kinase domain-like (MLKL) | Depletion of RIPK3 or MLKL blocks TNF-driven necroptosis and switches towards a delayed RIPK1 kinase-dependent apoptosis. Cell death & disease. 2014;5:e1004 |
| Mucin-5AC (MUC5AC) | MUC5AC protects pancreatic cancer cells from TRAIL-induced death pathways. International journal of oncology. 2013;42:887-93 |
| DNA-binding death effector domain-containing protein 2 (DEDD2) | Identification and characterization of DEDD2, a death effector domain-containing protein. The Journal of biological chemistry. 2002;277:7501-8 |
| Galactocerebrosidase (GALC) | A galactose-free diet enriched in soy isoflavones and antioxidants results in delayed onset of symptoms of Krabbe disease in twitcher mice. Molecular genetics and metabolism. 2010;100:234-40 |
| Nuclear pore complex protein Nup98-Nup96 (NUP98) | Graft-versus-leukemia (GVL) against mouse blast-crisis chronic myelogenous leukemia (BC-CML) and chronic-phase chronic myelogenous leukemia (CP-CML): shared mechanisms of T cell killing, but programmed death ligands render CP-CML and not BC-CML GVL resistant. Journal of immunology. 2011;187:1653-63 |
| Interferon regulatory factor 3 (IRF3) | Identification of TRAIL as an interferon regulatory factor 3 transcriptional target. Journal of virology. 2005;79:9320-4 |
| NADPH oxidase organizer 1 (NOXO1) | Death receptors 4 and 5 activate Nox1 NADPH oxidase through riboflavin kinase to induce reactive oxygen species-mediated apoptotic cell death. The Journal of biological chemistry. 2012;287:3313-25 |
| Protein S100-A6 (S100-A6) | Profiling of apoptotic changes in human breast cancer cells using SELDI-TOF mass spectrometry. Cellular physiology and biochemistry : international journal of experimental cellular physiology, biochemistry, and pharmacology. 2007;20:579-90 |
| Serine-protein kinase ATM (ATM) | TRAIL and guardian angel of genome integrity: ATM boards TRAIL blazer. Journal of cancer research and clinical oncology. 2011;137:1283-7 |
| Polypeptide N-acetylgalactosaminyltransferase 14 (GALNT14) | Development of immunohistochemistry assays to assess GALNT14 and FUT3/6 in clinical trials of dulanermin and drozitumab. Clinical cancer research : an official journal of the American Association for Cancer Research. 2010;16:1587-96 |
| Cyclin-dependent kinase 2 (CDK2) | Roscovitine sensitizes glioma cells to TRAIL-mediated apoptosis by downregulation of survivin and XIAP. Oncogene. 2004;23:446-56 |
| Histone deacetylase 2 (HDAC2) | HDAC2 attenuates TRAIL-induced apoptosis of pancreatic cancer cells. Molecular cancer. 2010;9:80 |
| Aryl hydrocarbon receptor (AHR) | Activation of the aryl hydrocarbon receptor sensitises human keratinocytes for CD95L- and TRAIL-induced apoptosis. Cell death & disease. 2012;3:e388 |
| Death receptor 4 (TNFRSF10A) | Death to the bad guys: targeting cancer via Apo2L/TRAIL. Apoptosis: an international journal on programmed cell death. 2005;10:35-51 |
| Death receptor 5 (TNFRSF10B) | Death to the bad guys: targeting cancer via Apo2L/TRAIL. Apoptosis: an international journal on programmed cell death. 2005;10:35-51 |
| Decoy receptor 2 (TNFRSF10D) | Death to the bad guys: targeting cancer via Apo2L/TRAIL. Apoptosis: an international journal on programmed cell death. 2005;10:35-51 |
| Osteoprotegerin (TNFRSF11B) | Death to the bad guys: targeting cancer via Apo2L/TRAIL. Apoptosis: an international journal on programmed cell death. 2005;10(1):35-51 |
| Caspase 8 (CASP8) | Death to the bad guys: targeting cancer via Apo2L/TRAIL. Apoptosis: an international journal on programmed cell death. 2005;10:35-51 |
| HOIL (RBCK) | Recruitment of the linear ubiquitin chain assembly complex stabilizes the TNF-R1 signaling complex and is required for TNF-mediated gene induction. Mol Cell. 2009 Dec 11;36:831-44. doi: 10.1016/j.molcel.2009.10.013 |
| Eukaryotic translation initiation factor 4E type 2 (EIF4E2) | Rocaglamide breaks TRAIL resistance in HTLV-1-associated adult T-cell leukemia/lymphoma by translational suppression of c-FLIP expression. Cell death and differentiation. 2011;18:362-70 |
